# Phylogeography of Eastern Grey Kangaroos, *Macropus giganteus*, Suggests a Mesic Refugium in Eastern Australia

**DOI:** 10.1371/journal.pone.0128160

**Published:** 2015-05-29

**Authors:** Brett A. Coghlan, Anne W. Goldizen, Vicki A. Thomson, Jennifer M. Seddon

**Affiliations:** 1 School of Biological Sciences, University of Queensland, St. Lucia, Queensland, Australia; 2 School of Veterinary Science, University of Queensland, Gatton, Queensland, Australia; BiK-F Biodiversity and Climate Research Center, GERMANY

## Abstract

Phylogeographic studies around the world have identified refugia where fauna were able to persist during unsuitable climatic periods, particularly during times of glaciation. In Australia the effects of Pleistocene climate oscillations on rainforest taxa have been well studied but less is known about the effects on mesic-habitat fauna, such as the eastern grey kangaroo (*Macropus giganteus*). The eastern grey kangaroo is a large mammal that is common and widespread throughout eastern Australia, preferring dry mesic habitat, rather than rainforest. As pollen evidence suggests that the central-eastern part of Australia (southeast Queensland and northern New South Wales) experienced cycles of expansion in mesic habitat with contraction in rainforests, and vice versa during glacial and interglacial periods, respectively, we hypothesise that the distribution of the eastern grey kangaroo was affected by these climate oscillations and may have contracted to mesic habitat refugia. From 375 mitochondrial DNA control region sequences from across the distribution of eastern grey kangaroos we obtained 108 unique haplotypes. Phylogenetic analysis identified two clades in Queensland, one of which is newly identified and restricted to a small coastal region in southern Queensland north of Brisbane, known as the Sunshine Coast. The relatively limited geographic range of this genetically isolated clade suggests the possibility of a mesic habitat refugium forming during rainforest expansion during wetter climate cycles. Other potential, although less likely, reasons for the genetic isolation of the highly distinct clade include geographic barriers, separate northward expansions, and strong local adaptation.

## Introduction

During past Pleistocene climatic cycles, many species survived in pockets of preferred habitat known as refugia, allowing them to persist through unfavourable local and regional climatic conditions [1, 2]. Climatic oscillations in Australia are thought to have been less extreme than those in the northern hemisphere, varying between cool/dry and warm/wet conditions rather than glacial/non-glacial [3–6]. Warm/wet cycles allowed rainforest habitat in eastern Australia to expand and conversely cool/dry cycles allowed mesic habitat to expand as rainforests contracted to refugial areas [7–10]. The boundaries of these rainforest refugia throughout the continent were constantly changing due to climate fluctuations [11]. In fact, phylogeographic patterns have been recognised for various rainforest species that support the hypothesis that multiple rainforest refugia occurred throughout the east coast of Australia during periods of climate fluctuation, allowing certain species to survive and diverge [12–15].

The identification of mesic barriers restricting gene flow among rainforest fragments, resulting in rainforest refugia, provides support for the expansion of mesic habitat [16]. The best-studied mesic-habitat barrier is the Black Mountain Barrier (BMB) in the Wet Tropics rainforest region of northern Queensland (QLD) in north-eastern Australia, which is considered a habitat barrier rather than purely a geographic (mountain) barrier [14, 17]. Several rainforest species spanning this region exhibit a north/south separation across the BMB, indicating connectivity early in the Pleistocene but subsequent separation by unsuitable habitat during the Last Glacial Maximum (LGM) that still persists today [15, 18, 19]. Examples of similar mesic-habitat barriers in other parts of QLD include the Burdekin Gap south of Townsville and the Broad Sound Barrier/St. Lawrence Gap, both in central-east QLD (e.g. [20, 21]).

**Fig 1:**
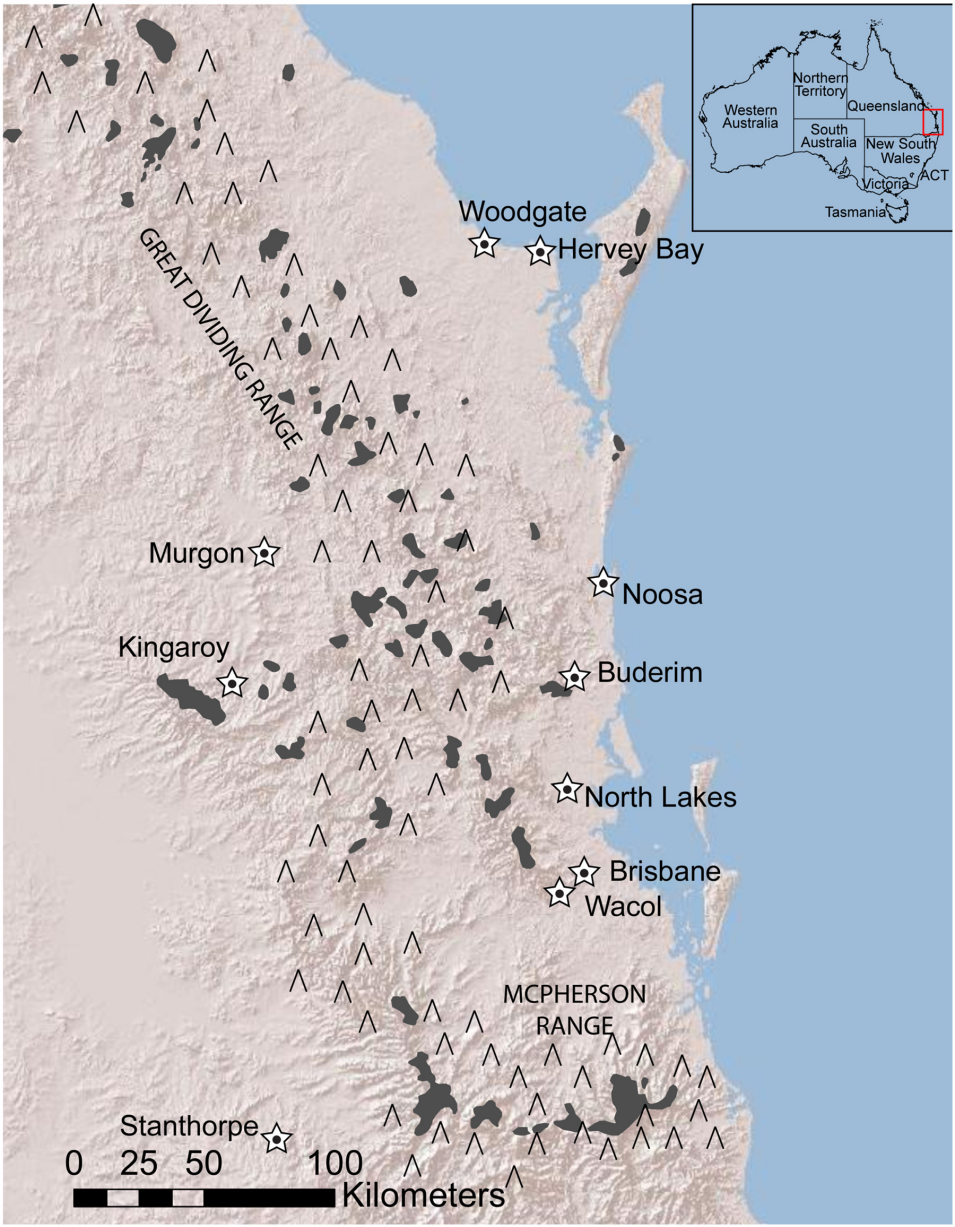
Contemporary temperate rainforest in southeast Queensland. Shaded areas indicate rainforest. Note that rainforest has been cleared in many areas since European colonisation. Cities/towns are represented by stars. Rainforest GIS layer package from Nauman [25] and locator map layer from Commonwealth of Australia (Geoscience Australia) [26]. ACT—Australian Capital Territory; ∧– Great Dividing Range.

In contrast to these studies of rainforest refugia, there has been little research conducted on the effects of refugia on mesic, open-woodland species in Australia (but see [22–24]). It is likely that the mesic habitats, such as dry and open woodlands that were previously barriers to rainforest species, may have acted as refugia for mesic-adapted species when tropical and subtropical rainforests expanded during warm/wet climate cycles. Here we test this hypothesis in southeast QLD (including the areas surrounding Brisbane and the Sunshine Coast region north of Brisbane) where there is a modified mosaic of closed rainforest interspersed with drier habitat along, and to the east (coastal side) of, the Great Dividing Range (GDR; Fig 1) [16, 25, 26]. Estimates of rainforest species distributions indicate that until recently rainforests were contiguous across such high altitudes as the GDR [16]. In addition, pollen records support repeated cycles of expanding/contracting rainforest and mesic forest vegetation in north-eastern New South Wales (NSW; located directly south of QLD) and southeast QLD during the Pleistocene [8, 9, 27]. Furthermore, rainforest species in the region show genetic patterns that are consistent with their having had such restricted ranges during the cool, dry period of the LGM [16]. However, there is little direct evidence for refugia and expansion patterns in mesic-habitat species in the area.

The eastern grey kangaroo (*Macropus giganteus*) is useful for examining the hypothesis that mesic-habitat refugia occurred in southeast QLD since it is widespread and common (non-threatened), prefers mesic habitat, and avoids rainforests [28]. Although the kangaroos’ large size makes them difficult to trap, their abundance facilitates non-invasive sampling from faecal material. Eastern grey kangaroos range throughout QLD and NSW, as well as other states in the eastern part of Australia including Victoria (VIC), parts of Tasmania (TAS), and the extreme eastern part of South Australia, and prefer open-woodland habitat that provides adequate grass cover for forage and shade for rest during the heat of the day [28–31]. The eastern grey kangaroo is a vagile species that does not appear to exhibit genetic isolation-by-distance at a widespread scale, suggesting contemporary gene flow occurs across the wider species’ distribution [23]. A similar species, the red kangaroo (*Macropus rufus*), occupies drier habitat than the eastern grey kangaroo and exhibits significantly higher diversity across its range and structure that correlates with topography, suggesting regions of higher altitude may have provided refuges during past climate cycles [32]. A previous phylogeographic study on eastern grey kangaroos, based on the mitochondrial DNA control region (mtDNA *CR*), found clear differentiation between northern individuals in inland QLD and southern individuals in NSW, VIC, and TAS, suggesting a likely northwards migration from a southern origin possibly as environmental amelioration occurred after past climate change events [23], however this study lacked samples from southern and coastal (eastern) QLD. Based on these prior studies, we hypothesise that the distribution of eastern grey kangaroos was affected by the climate oscillations of previous eras. Thus, this paper has two aims. The first is to investigate possible refugial areas by examining the genetic variation in eastern grey kangaroos within the mosaic of mesic and rainforest habitats at a regional scale in southeast QLD to investigate the evidence for mesic refugia, while the second is to investigate species-wide phylogeographic patterns by filling large gaps in existing, yet patchy, range-wide datasets to more accurately determine how southeast QLD kangaroos fit into the wider phylogeographic patterns exhibited by this species.

## Materials and Methods

### Sample collection

**Fig 2:**
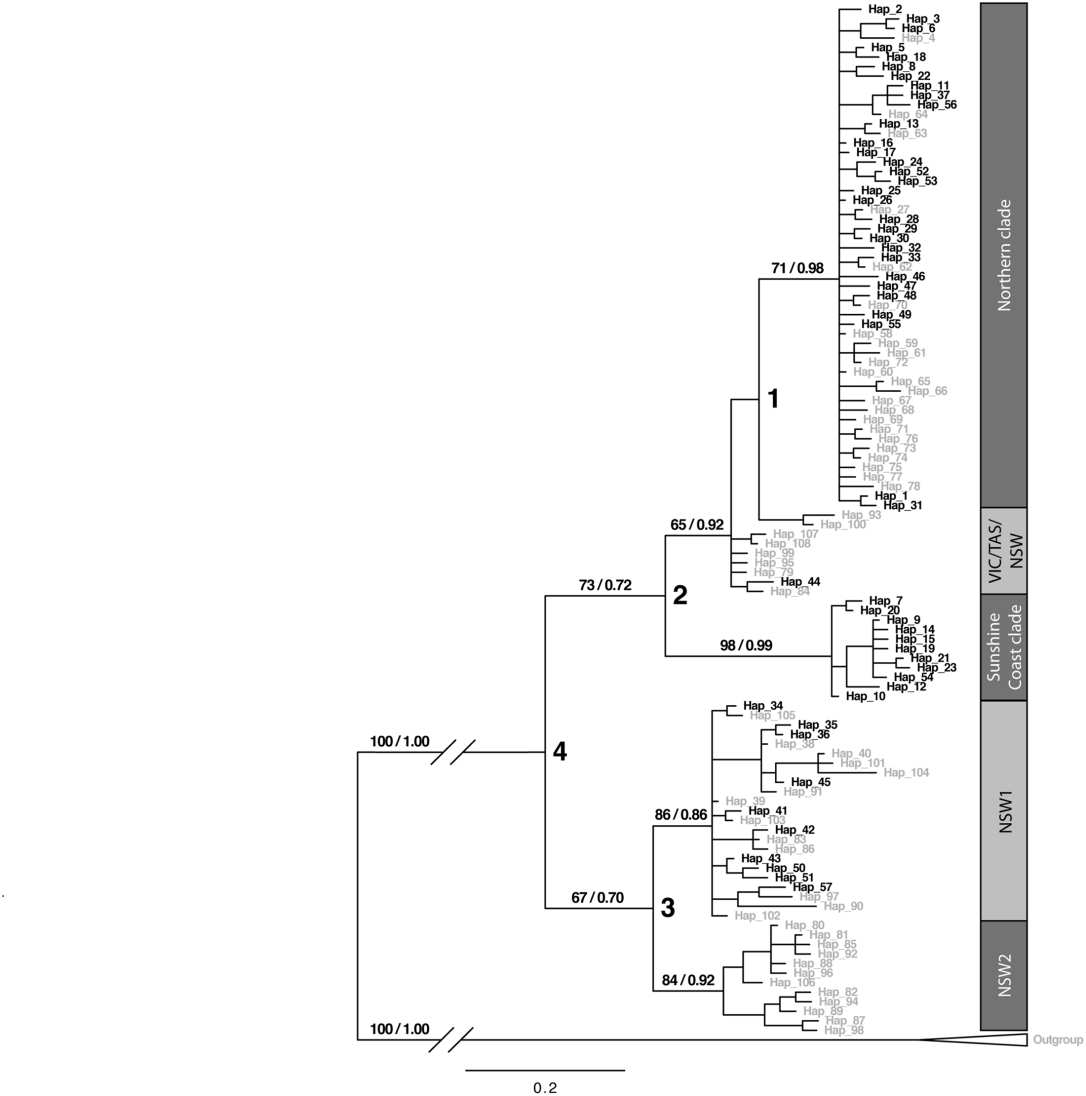
Bayesian inference phylogenetic tree of mtDNA control region for *M*. *giganteus*. Numbers on branches represent ML bootstrap support/Bayesian posterior probabilities. Support is only shown on major clades (Bayesian posterior probability > 0.5). Numbers at nodes correspond to splitting events discussed in the text. Haplotypes in black indicate new haplotypes from this study, while haplotypes in grey represent sequences from Zenger *et al*. [23].

A total of 317 faecal and tissue samples were collected from 39 sites throughout QLD and NSW. Faecal samples were collected fresh and either placed in paper bags, air-dried and stored at -20°C, or placed in sterile containers and frozen at -20°C as soon as feasible [33]. We chose to primarily use non-invasive sampling techniques due to sampling within the semi-urban environment, cost, and time-effectiveness that precluded getting samples from kangaroo harvesters or from trapping a large mammal such as the eastern grey kangaroo. Although we cannot confirm that every faecal sample came from a different individual, when possible we observed an individual kangaroo defecating and collected that sample. Samples from Elanda Point and Sundown National Park were collected from different individual kangaroos, identified as part of other behavioural studies. Our phylogenetic analyses do not rely on haplotype frequency and thus the presence of multiple samples from a single individual will not have affected inferences from phylogenetic analyses. Tissue samples were opportunistically taken as ear clips from road-killed or otherwise deceased kangaroos in QLD only and were stored in 100% ethanol at -20°C. It was not possible to obtain samples from harvested kangaroos, as kangaroos are not harvested in the semi-urban area that was the main focus of our study. In addition to 41 samples collected in 2008 and the 215 collected in 2012 for this study, 58 eastern grey kangaroo mtDNA sequences were obtained from GenBank from a previous phylogeographic study (GenBank accession numbers AF443122.1–AF443172.1, EF555437.1–EF555443.1) [23] and three sequences from western grey kangaroos (*Macropus fuliginosus*; AF443173.1–AF443175.1) were used as an outgroup (Fig 2; S1 File).

#### Ethics Statement

The collection protocol was approved by the University of Queensland Native/Exotic Wildlife and Marine Animals ethics committee and collection permits WISP10606612 and WITK10606712 were obtained from Queensland’s Department of Environment and Resource Management. No permits were required for faecal sample collection in New South Wales. This study did not involve any endangered or protected species.

### DNA extraction

DNA was extracted from the majority of faecal samples using the QIAmp DNA Stool Mini Kits (QIAGEN, Chadstone Centre) with a 200 μL elution, and 22 samples were extracted using the ISOLATE Fecal DNA Kit (Bioline, Alexandria) with a 100 μL elution. DNA extractions from tissue samples followed a Proteinase K digestion and a salting-out procedure similar to Nicholls *et al*. [34].

### mtDNA sequencing

We sequenced 570 bp of the mtDNA *CR* using marsupial-specific primers H16498M and L15999M [35]. We used a 10 μL polymerase chain reaction (PCR) mixture comprised of 1x PCR buffer (Qiagen, Chadstone Centre), 1.5 mM MgCl_2_, 0.25 μM of each primer, 0.15 mM each dNTP, and 0.5 U HotStar Taq DNA polymerase (Qiagen, Chadstone Centre). PCR reactions were run with the following conditions: initial denaturation at 94°C for 15 minutes, 40 cycles of denaturation at 94°C for 30 seconds, annealing at 55°C for 30 seconds, and extension at 72°C for 1 minute, followed by a final annealing step at 50°C for 5 minutes and a final extension at 72°C for 10 minutes.

PCR products were purified by adding 5 U exonuclease I, *Escherichia coli* (Fermentas, Scoresby) and 0.5 U shrimp alkaline phosphatase (Promega, Hawthorn) and incubating at 37°C for 30 minutes followed by an enzyme inactivation step of 80°C for 15 minutes. Following purification, we performed a cycle sequencing reaction by using a 12 μL reaction mixture with 3.0 μL 5x sequencing buffer, 0.5 μL primer, and 1.0 μL BigDye v3.1 (Applied Biosystems, Mulgrave). Both strands were sequenced using the respective PCR primers. The products were precipitated following the manufacturer’s protocol of using EDTA, sodium acetate, and ethanol. Capillary electrophoresis was undertaken by the Animal Genetics Laboratory (AGL) at the University of Queensland, Gatton, on an ABI 3730xl or 3130xl Genetic Analyzer (Applied Biosystems, Mulgrave). We attempted to examine nuclear markers but were unsuccessful due to low microsatellite amplification rates, likely a result of low quality DNA in the faecal samples.

### Analyses

Sequences were assembled and checked for errors in ChromasPro (Technelysium, South Brisbane) and aligned using Clustal W in MEGA v5.1 [36, 37]. Nucleotide diversity (*π*), haplotype diversity (*h*), Tajima’s *D* [38], and Fu and Li’s F* [39] were calculated in DnaSP v5.10.1 [40] using the 256 sequences obtained in this study and 61 GenBank sequences to form a complete data set (n = 317). Fu’s *F*_*S*_ [41] for each clade and respective *P*-values were calculated in Arlequin v3.5.1.2 [42] using 10,000 simulated samples.

A maximum likelihood (ML) tree was constructed in MEGA v5.1 [36] and Bayesian phylogenetic analysis was conducted using MrBayes v3.2.1 [43]. Using jModelTest v2.1.1 [44, 45], the best-fit nucleotide substitution model for phylogenetic analysis was a Tamura-Nei model (TrN) + gamma distribution [46] with gamma shape parameter 3.1020. This model was used in the maximum likelihood analysis while the generalised time-reversible (GTR) + gamma distribution model was used in MrBayes, given that MrBayes does not provide TrN as an available substitution model.

We ran the Bayesian analysis for 5,000,000 generations to obtain a standard deviation of split frequencies less than 0.01 and discarded 25% as burn-in. As the large number of sequences proved computationally intensive, maximum likelihood and Bayesian phylogenetic analyses were only conducted on a smaller set of unique haplotypes, generated in DnaSP. The tree was edited for publication using FigTree v1.4.0 [47]. We also used MrBayes to determine if the ancestry of nodes 2, 3, and 4 from the resulting phylogenetic tree was northern, southern, or of outgroup (western grey kangaroo) origin. Median joining haplotype networks were created for all unique *M*. *giganteus* haplotypes, plus the Sunshine Coast clade separately using Network v4.6.1.3 (www.fluxus-engineering.com [48, 49]). Arlequin v3.5.1.2 [42] was used to estimate demographic expansion parameters of the Sunshine Coast clade through mismatch distributions with 100 bootstrap replicates.

## Results

A total of 256 samples were successfully sequenced (S1 File) and aligned (S2 File) with the 61 sequences from Zenger *et al*. [23]. Among the full dataset (n = 317), there were 111 unique haplotypes with 141 polymorphic sites, of which 121 were parsimony informative. New haplotypes were uploaded to GenBank (KF316482–KF316538).

**Fig 3:**
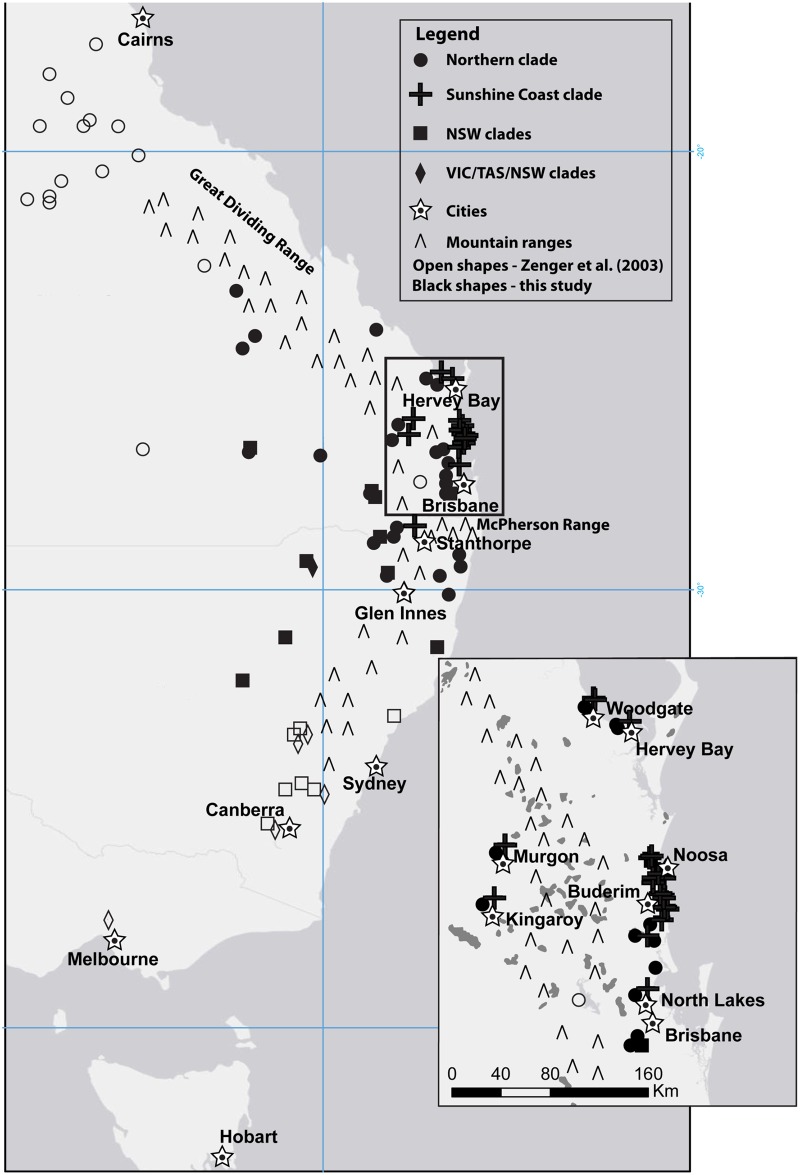
Sampling and haplotype locations. Map showing location and haplotype designations of each clade according to the phylogenetic tree. Circles represent the Northern clade, diamonds represent the VIC/TAS/NSW clade, crosses represent the Sunshine Coast clade, squares represent both NSW 1 and NSW 2 clades, and stars represent cities. The open shapes represent haplotypes from Zenger *et al*. [23] and solid black coloured shapes represent new haplotypes sampled in this paper. Inset is the Sunshine Coast region; shading in inset indicates contemporary temperate rainforest.

**Fig 4:**
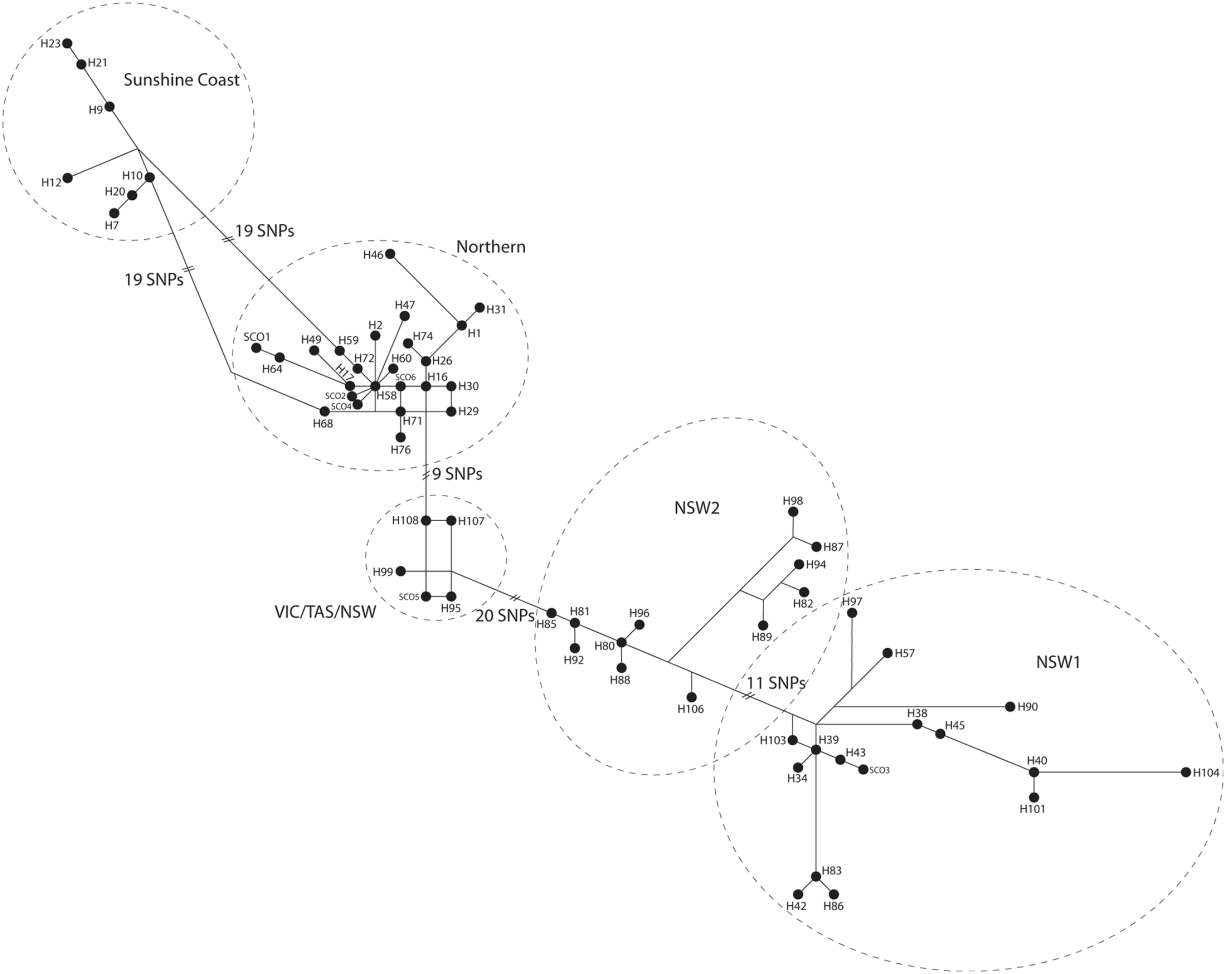
Haplotype network of *M*. *giganteus*. The haplotype numbers are the same as in the phylogenetic tree and the dashed circles correspond to clades from Fig 2. Nodes labelled “SCO” indicate multiple haplotypes collapsed into a single node by star contraction in Network. SCO1 = H_11, H_37, H_56; SCO2 = H_8, H_13, H_22, H_24, H_27, H_28, H_32, H_52, H_53, H_55, H_63, H_67; SCO3 = H_50, H_51; SCO4 = H_33, H_62, H_69; SCO5 = H_44, H_79, H_84; SCO6 = H_3, H_4, H_5, H_6, H_18, H_65, H_66.

Both ML and Bayesian methods of phylogenetic analysis consistently reconstructed five clades, although with differing support levels (Fig 2). Importantly, we identified a strongly supported, highly distinct clade that was restricted to southeast QLD, predominately the area near the Sunshine Coast (hereafter termed the Sunshine Coast clade). The other four clades were less strongly supported but were consistent with those identified in Zenger *et al*. [23]: 1) a Northern clade with individuals from QLD and northern NSW extending south to Glen Innes (NSW) and Moree (NSW); 2) a clade consisting of individuals from VIC, TAS, and NSW (VIC/TAS/NSW) was identified on the ML tree, however these individuals did not form a clade on the Bayesian tree but rather were sister taxa to the monophyletic Northern clade; 3) and 4) two clades consisting mainly of NSW individuals, with a few haplotypes from southern QLD (Fig 3). Our network analysis identified five groups consistent with the clades identified in our Bayesian inference tree (Fig 4). The geographical distribution of the clades is shown in Fig 3; however, the two NSW clades are represented as one in the figure due to lack of detailed sampling locations available from Zenger *et al*. [23] leading to an inability to determine exact locations within NSW for a few of the samples. Bayesian posterior probability support in the Bayesian inference tree was strongest for the Sunshine Coast clade (0.99) and the Northern clade (0.96) and lower for the other clades, ranging from 0.82 to 0.89 (Fig 2). The topology of major clades in the ML tree was identical to the Bayesian tree with the ML tree containing some clades with lower bootstrap values, although all clades had bootstrap values greater than 65 (Fig 2). Our analysis to determine the origin of nodes 2, 3, and 4 resulted in the highest probabilities of P(south) = 0.98 for node 2 [P(north) = 0.018; P(outgroup) = 0.0034], P(south) = 0.99 for node 3 [P(north) = 0.00099; P(outgroup) = 0.00034], and P(outgroup) = 0.99 for node 4 [P(north) = 0.00037; P(south) = 0.00056].

The Tajima’s *D* and Fu and Li’s F* statistics for all five major clades were non-significant (Table 1), suggesting population stability. However Fu’s *F*_*S*_ values were significant for the Northern clade, the NSW clades combined, each of the NSW 1 and NSW 2 clades separately, as well as the species as a whole (Table 1), indicating recent population expansion for these groups. Haplotype diversity was high (*h* > 0.5) for all clades, with nucleotide diversity also high (*π* > 0.5%) for all clades except the Sunshine Coast clade (*π* = 0.402%; Table 1).

**Table 1:** Diversity statistics for clades identified in Bayesian phylogenetic analysis.

Clade	No. of haplotypes	*π*	*h*	*k*	Tajima’s *D*	*P*-value	Fu and Li’s F*	*P*-value	Fu’s *F*_*S*_	*P*-value
**Northern**	54	0.010	0.960	5.833	-1.223	> 0.10	-2.129	> 0.05	-23.887	0.003^a^
**Sunshine Coast**	11	0.004	0.644	2.086	-0.672	> 0.10	-0.403	> 0.10	-7.323	0.085
**VIC/TAS/NSW**	10	0.010	1.000	6.156	-0.608	> 0.10	-0.378	> 0.10	-1.931	0.099
**NSW 1 and 2 combined**	35	0.018	0.960	10.852	-0.785	> 0.10	-1.420	> 0.10	-24.038	< 0.001^a^
**NSW 1**	23	0.012	0.940	7.044	-0.930	> 0.10	-1.924	> 0.10	-20.140	< 0.001^a^
**NSW 2**	12	0.012	1.000	7.682	0.038	> 0.10	0.060	> 0.10	-4.469	0.017^a^
***M*. *giganteus* (total)**	108	0.041	0.960	20.304	0.351	> 0.10	-0.353	> 0.10	-23.794	< 0.001^a^

Diversity statistics of the supported clades using the complete data set (n = 317). Tajima’s *D* and Fu and Li’s F* were not significant for any clade. Some statistics were not calculated for *M*. *fuliginosus* due to the low sample size (n = 3). *π*—nucleotide diversity, *h*—haplotype diversity *k*—average number of nucleotide differences within each clade.

^a^ Indicates significance (< 0.02) of Fu’s *F*_*S*_.

**Fig 5:**
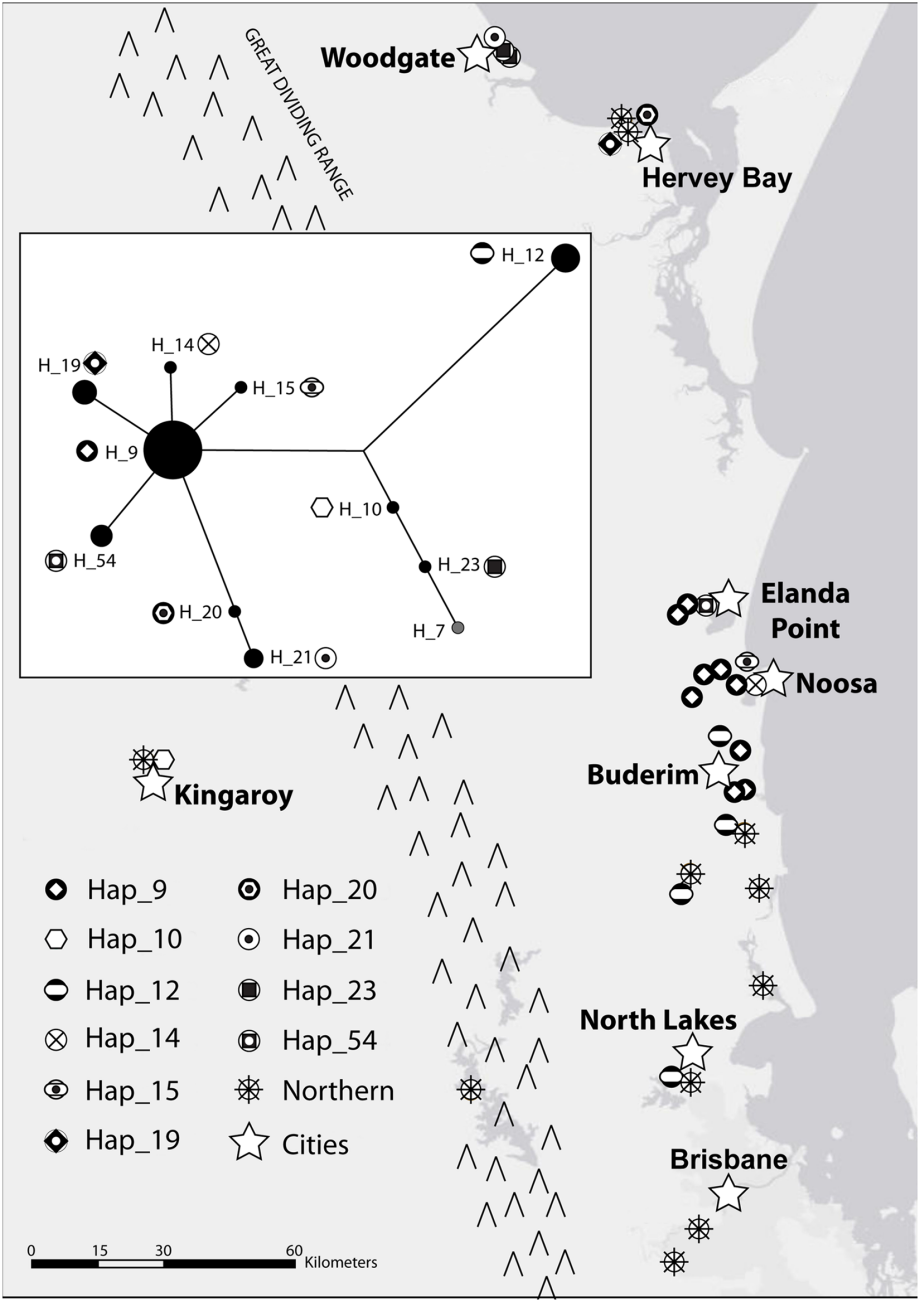
Distribution of haplotypes in the Sunshine Coast region. Map showing the distribution of Sunshine Coast clade haplotypes and Northern clade haplotypes in the Sunshine Coast region. Hap_7 of the Sunshine Coast clade (not pictured) was located ~170 km SW of Brisbane in Stanthorpe, QLD. A single individual with a haplotype from the NSW clade (not labelled) was found at Elanda Point, ~18 km NNW of Noosa. Inset is a haplotype network of the Sunshine Coast clade. Node size corresponds to relative number of individuals with that haplotype and distance between nodes corresponds to number of mutations.

Monophyly of the newly discovered Sunshine Coast clade had high support values in both of the mtDNA *CR* phylogenetic trees (Fig 2) and this clade was genetically divergent from both the Northern clade (5%) and NSW clades (7%). The Sunshine Coast clade was comprised of eleven haplotypes, with the network showing one common haplotype with four closely related haplotypes and six more distant haplotypes (Fig 5 inset). All haplotypes in this clade except one occurred within a 23,000-km^2^ area in south-eastern QLD along and inland from the Pacific coast, between Hervey Bay (to the north), North Lakes (to the south), Kingaroy (to the west), and the Pacific Ocean (to the east; Fig 3; Fig 5). A single individual with a Sunshine Coast haplotype was found in Stanthorpe, QLD (~170 km southwest of Brisbane), while all other individuals from Stanthorpe (n = 6) belonged to the Northern clade.

## Discussion

Here we identify a mtDNA clade that was predominantly restricted to the Sunshine Coast region of QLD (plus areas just to the north and west of there) and was genetically divergent from clades in surrounding regions currently connected by suitable habitat. The limited geographic range of the Sunshine Coast clade, together with its clear divergence from other nearby populations, is consistent with a population of eastern grey kangaroos having survived in a mesic refugium for long periods of time. The repeated cycles of alternating expansion and contraction of rainforest and mesic forest vegetation in northern NSW and southeast QLD during the Pleistocene [8, 9, 27] may have been the driving force behind such a refugium. Genetic divergences in two other species with similar habitat preferences to the eastern grey kangaroo, the sugar glider (*Petaurus breviceps*) [50] and the squirrel glider (*Petaurus norfolcensis*) [51], also showed parallels with climatic cycles. These gliders show evidence of range contractions during time periods when climate hindered their ideal habitat, but range expansions when the climate supported their ideal habitat. In addition, koalas (*Phascolarctos cinereus*) show evidence of mtDNA divisions between coastal and inland populations, consistent with closed rainforests at higher altitude having acted as a wet habitat barrier to the preferred mesic habitat of this species [24].

Although refugial populations characteristically undergo reductions in population size, we found contradictory evidence for a bottleneck or post-bottleneck expansion in the Sunshine Coast clade. When compared to the eastern grey kangaroo species as a whole, this clade exhibits low nucleotide diversity but medium-high haplotype diversity (Table 1), which can be indicative of a bottleneck followed by population growth [52]. Similarly indicative of a bottleneck, the majority of Sunshine Coast individuals exhibited a single haplotype (Hap_9; Fig 5 inset; although it should be noted this may reflect sampling bias), with the area around Noosa (QLD) showing the least amount of incursion from haplotypes that were assigned to other clades (Fig 3). Our high concentration of samples from near Elanda Point and Noosa might explain the apparently high frequency of Hap_9. Equally intensive sampling in other regions of the Sunshine Coast may identify additional common haplotypes. Furthermore, with our non-invasive sampling techniques we cannot say for certain if all samples and sequences came from different individuals although the samples from Elanda Point were all from identified individuals. However, additional statistics such as Tajima’s *D*, Fu and Li’s F*, and Fu’s F_S_ that can test for population bottlenecks were non-significant (Table 1). Hence, it is possible but not conclusive that the Sunshine Coast population has remained at a long-term low effective population size. For the eastern grey kangaroo species as a whole, the high nucleotide and high haplotype diversity suggests a large and stable population [52], as would be expected for a species with its large body size and natural history [53]; the 2011 estimated population of eastern grey kangaroos in Queensland was nearly 10.8 million [54].

An alternative explanation for genetic isolation is an effect of physical or topographical barriers to gene flow. However, there are no obvious physical or topographical barriers preventing gene flow immediately to the north, south, and west of the Sunshine Coast region. At a wider scale, geographic barriers in eastern Australia including the GDR, Brisbane River, and McPherson Range, appear not to have restricted dispersal in eastern grey kangaroos of the Sunshine Coast and Northern clades. Hence, the genetic isolation of the Sunshine Coast clade is more consistent with historical isolation due to unsuitable habitat than with clear geographic barriers.

Haplotypes from the Northern and Sunshine Coast clades currently overlap in regions surrounding the Sunshine Coast (Murgon, Kingaroy, Woodgate, Hervey Bay, Buderim, and North Lakes; Fig 3; Fig 5). The genetic isolation of these clades suggests that this geographic overlap was not present, or at least not as widespread, during the period when the Sunshine Coast region was isolated. There has been significant anthropogenic habitat modification such as urbanisation and forest clearing in coastal areas of eastern Australia, including the Sunshine Coast region, in recent times. This modification will have increased kangaroo-favourable habitat and allowed kangaroos to expand outwards from the Sunshine Coast, thereby increasing contact with individuals from the Northern clade.

Another explanation for the presence of two distinct clades of eastern grey kangaroos in northern Australia is that there may have been two separate incursions of eastern grey kangaroos into QLD from southern Australia. Zenger *et al*. [23] proposed that eastern grey kangaroos in QLD originated from a single northward migration from southern regions (divergence between NSW and QLD clades at node 4 in Fig 2). Our results are compatible with a separate migration into southeast QLD, with the Sunshine Coast the remnant of this migration or expansion event (Sunshine Coast clade divergence at node 2 in Fig 2). The low probability of a northern origin for nodes 2, 3, and 4 supports the southern origin of the species. The high probability of a southern origin for node 2 [P(south) = 0.98] specifically suggests that the individuals in the Sunshine Coat clade were not a recent split from a northern population and that they had migrated from the south. Under this scenario, it is possible that the northern migration suggested by Zenger *et al*. [23] was actually a separate, possibly subsequent, migration northward into QLD (the Northern clade divergence at node 1 in Fig 2), with eastern grey kangaroos at that time successfully colonising a widespread part of eastern QLD. However, we did not identify any haplotypes of the Sunshine Coast clade along a possible expansion route, either inland or along the coast, nor in any potential source population in southern Australia, and so we consider this explanation unlikely. Further sampling required would be required to test it thoroughly.

It is also possible that the Sunshine Coast clade represents a population of kangaroos that underwent strong local adaptation, possibly to soil type. However, our data are insufficient to address this hypothesis and sampling of soils and vegetation as well as more genetic data, particularly from autosomal markers, would be necessary to confirm or rule out local adaption.

In our phylogeographic trees the nodes of southern lineages in NSW appear more basal, providing support for the conclusion of Zenger *et al*. [23] that eastern grey kangaroos originated in southern Australia and migrated northwards. However, our study identified that the Northern clade extends farther south into NSW than previously thought (Fig 3) [23]. The area of mixing between the Northern and NSW 1 clades appears to centre on the QLD/NSW border with individuals from both clades having been sampled in Glen Innes (NSW), Moree (NSW), Sundown National Park (QLD), and Wacol (QLD). The cause of the original separation between the Northern and NSW clades remains unclear [23]. Another finding is the unusual placement of the VIC/TAS/NSW clade as sister to the Northern clade. There are no contemporary records of translocations of kangaroos from the mainland to Tasmania [55] and the nodes of most other haplotypes from the southern part of the eastern grey kangaroos’ range not a part of the VIC/TAS/NSW clade are basal to the Northern clade. The origin of this VIC/TAS/NSW clade also remains unclear.

The presence of similar or identical haplotypes in geographically distant sampling locations suggests gene flow has occurred over long distances in the eastern grey kangaroos, a pattern also seen in the previous study [23]. Given that the maximum observed dispersal distance of an eastern grey kangaroo is about 17 km [56], we cannot completely rule out the possibility that a few of the instances of apparent long-distance dispersal events in our data were caused by orphaned kangaroos that were taken into care being released at a distance from their capture location. Possible examples of such cases include one individual sampled from Elanda Point in the Sunshine Coast region that clustered with NSW samples in the phylogenetic analysis, one individual from Roma, QLD (~430 km WNW of Brisbane) that clustered with a NSW clade, and one individual from Stanthorpe, QLD that clustered with the Sunshine Coast clade. It is always possible though that other individuals with haplotypes from our Sunshine Coast clade occur in other areas but our sampling process simply failed to obtain those individuals due to sampling bias, just as it is possible that our sampling process missed individuals from the Northern clade that may occur in the Sunshine Coast region or in New South Wales and vice versa.

In conclusion, we identified a divergent Sunshine Coast clade of eastern grey kangaroos that most likely resulted from a mesic-habitat refugium in southeast QLD. However confirming the presence of a refugium in this region and determining its extent requires geographically fine-scale studies of other mesic taxa as well as historical vegetation modelling. With regards to the eastern grey kangaroo, generating additional genetic data based on nuclear loci will be necessary to assess whether the Sunshine Coast clade warrants designation as a management or evolutionarily significant unit. Nuclear markers may also establish whether the divergence patterns that we identified are influenced by the species’ male-biased dispersal and identify any recent genetic admixture between animals from different mtDNA clades [23, 56].

## Supporting Information

S1 File Information for each sample collected. Sample name, location, haplotype, clade, GenBank accession number, and source for all *M*. *giganteus* samples included in this study.
(XLSX)

S2 File Sequence alignment. The alignment of all 108 *M*. *giganteus* haplotypes included in our analysis as well as information of haplotype frequencies. Invariable sites have been removed from the alignment.
(TXT)
